# Supplementary figures and images for: The effectiveness of arbuscular mycorrhizal fungal species (Funneliformis mosseae, Rhizophagus intraradices, and Claroideoglomus etunicatum) in the biocontrol of root and crown rot pathogens, Fusarium solani and Fusarium mixture in pepper
Source: PeerJ. 2025 Jan 16;13:e18438. doi: 10.7717/peerj.18438 (PMC11742253; doi:10.7717/peerj.18438)

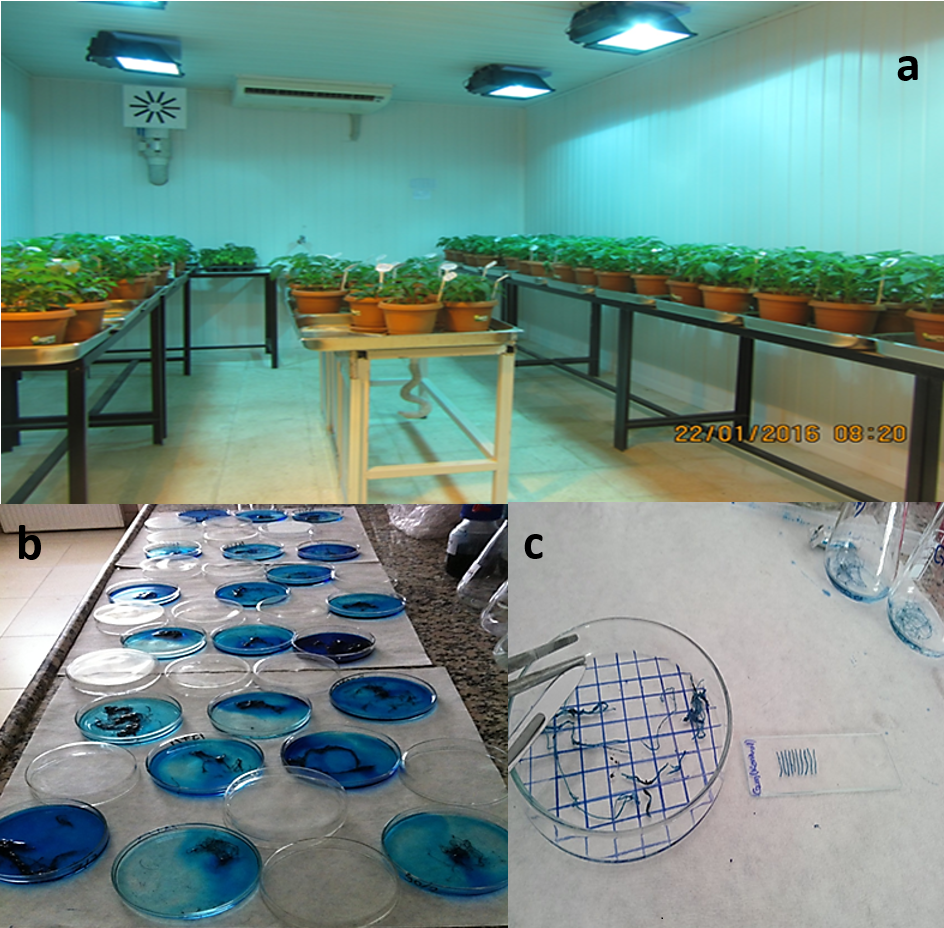

Supplement: Supplemental Information 11 [file peerj-13-18438-s011.png]

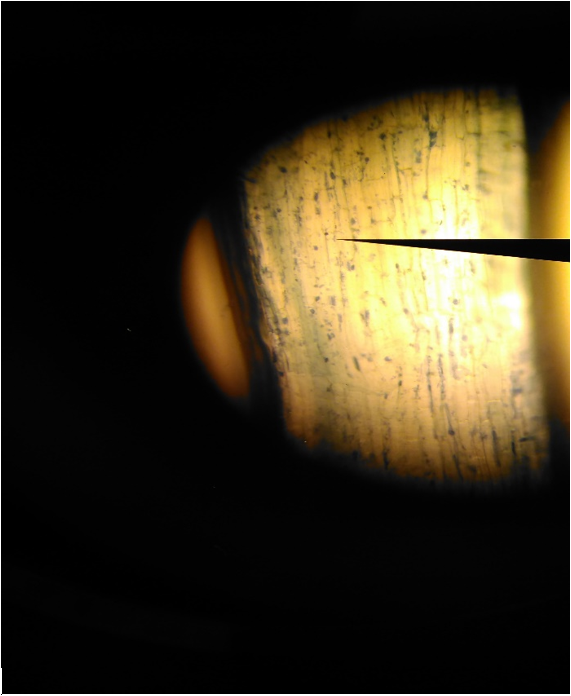

Supplement: Supplemental Information 12 [file peerj-13-18438-s012.png]

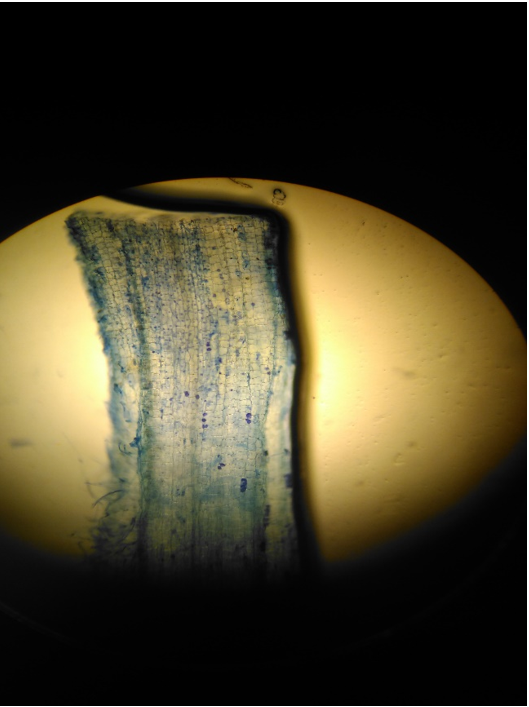

Supplement: Supplemental Information 13 [file peerj-13-18438-s013.png]

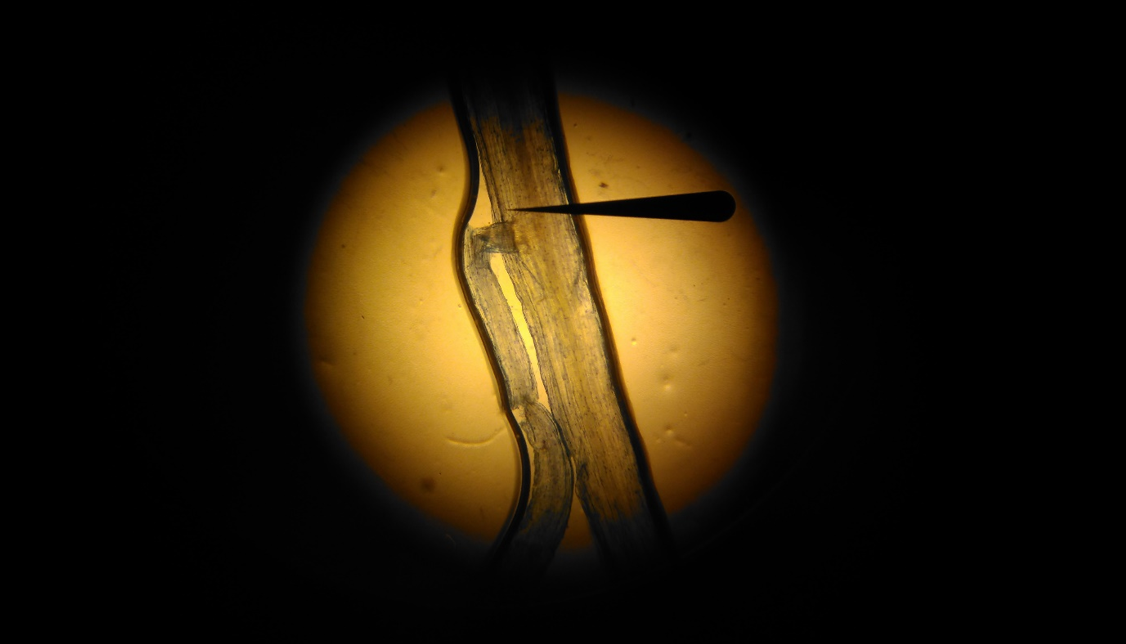

Supplement: Supplemental Information 14 [file peerj-13-18438-s014.png]

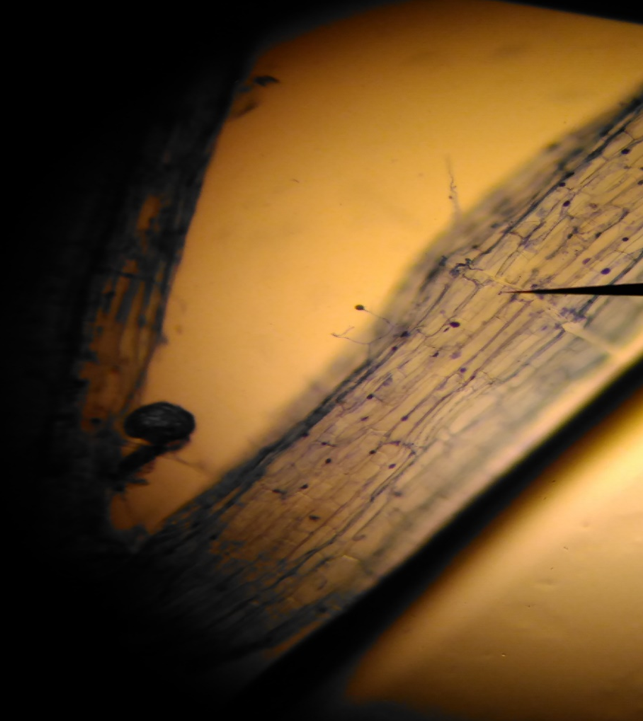

Supplement: Supplemental Information 15 [file peerj-13-18438-s015.png]

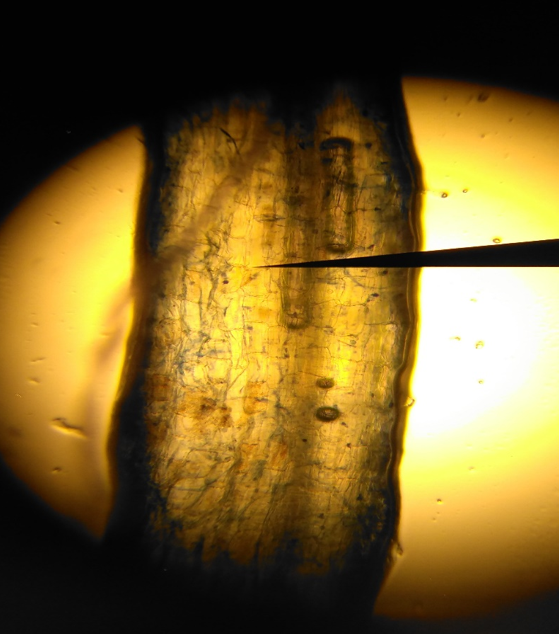

Supplement: Supplemental Information 16 [file peerj-13-18438-s016.png]

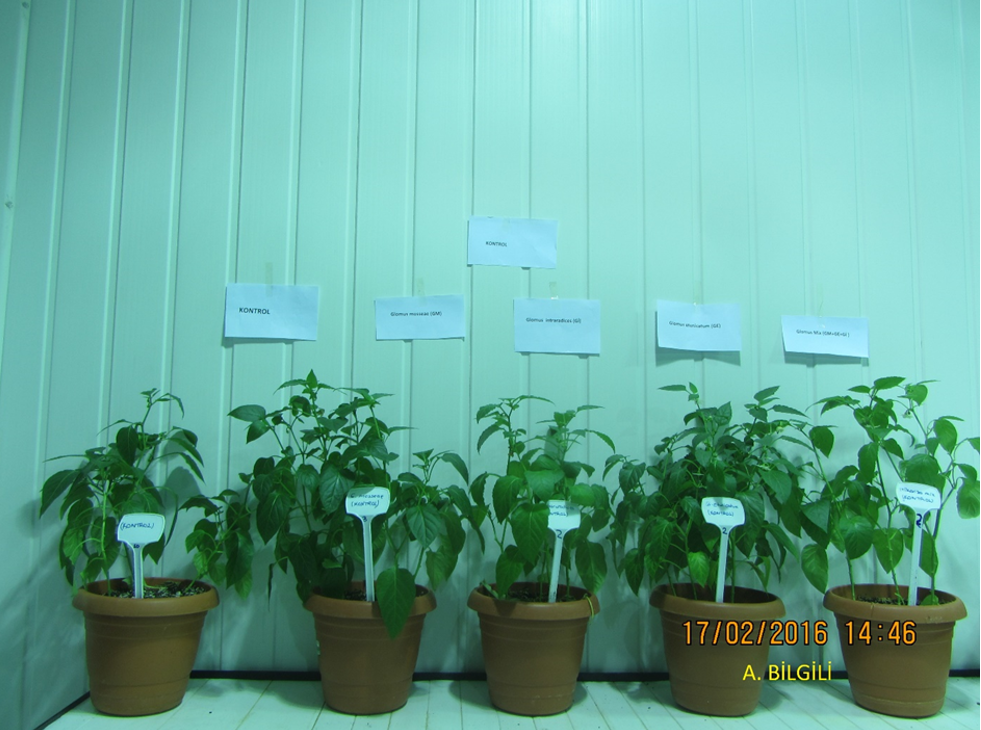

Supplement: Supplemental Information 17 [file peerj-13-18438-s017.png]

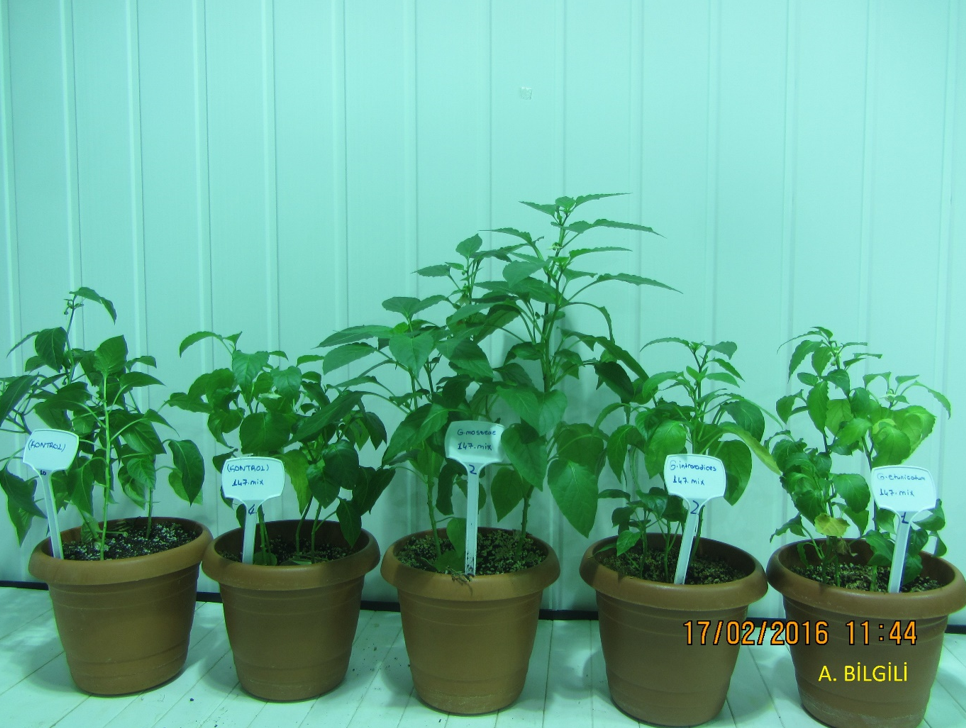

Supplement: Supplemental Information 18 [file peerj-13-18438-s018.png]

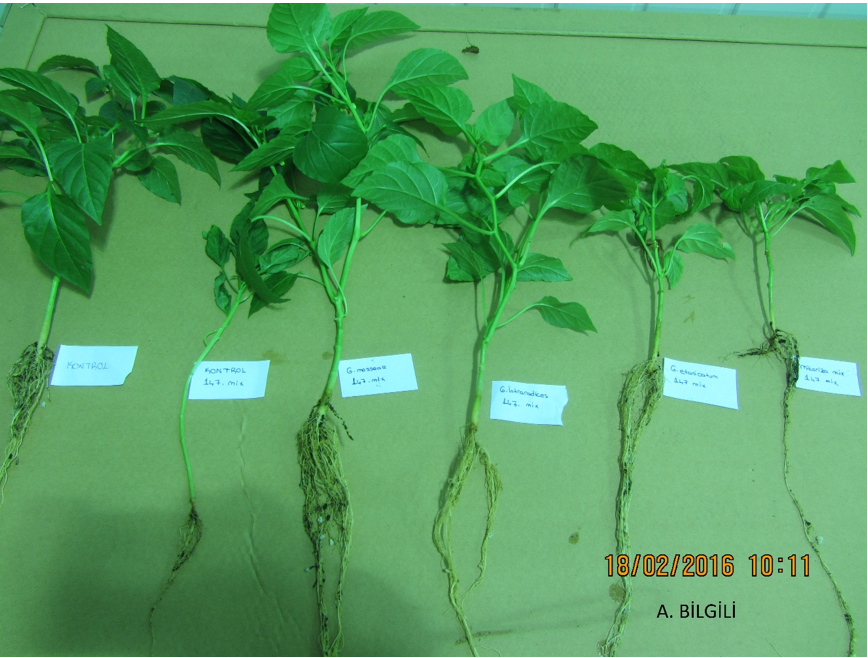

Supplement: Supplemental Information 19 [file peerj-13-18438-s019.png]

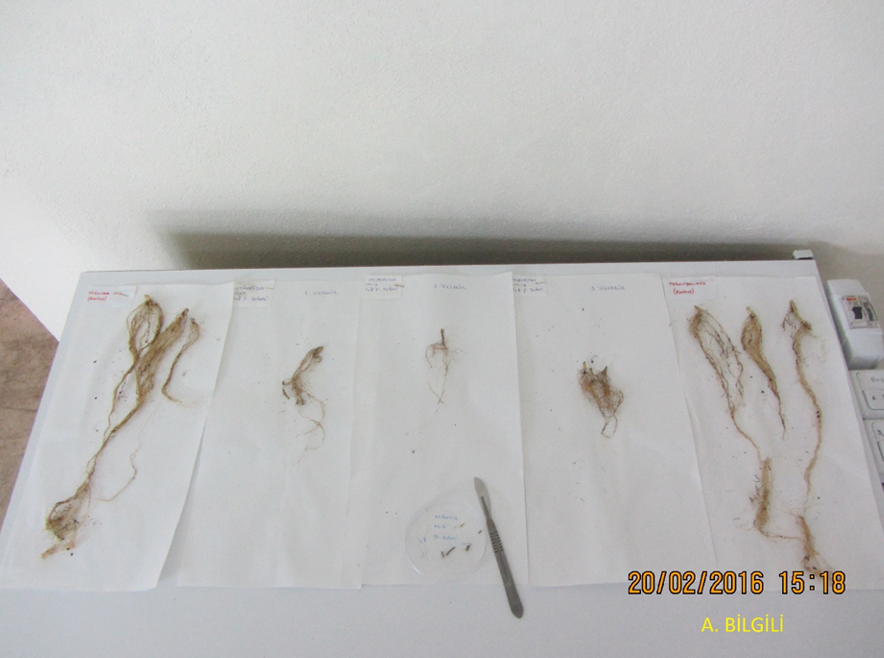

Supplement: Supplemental Information 20 [file peerj-13-18438-s020.png]

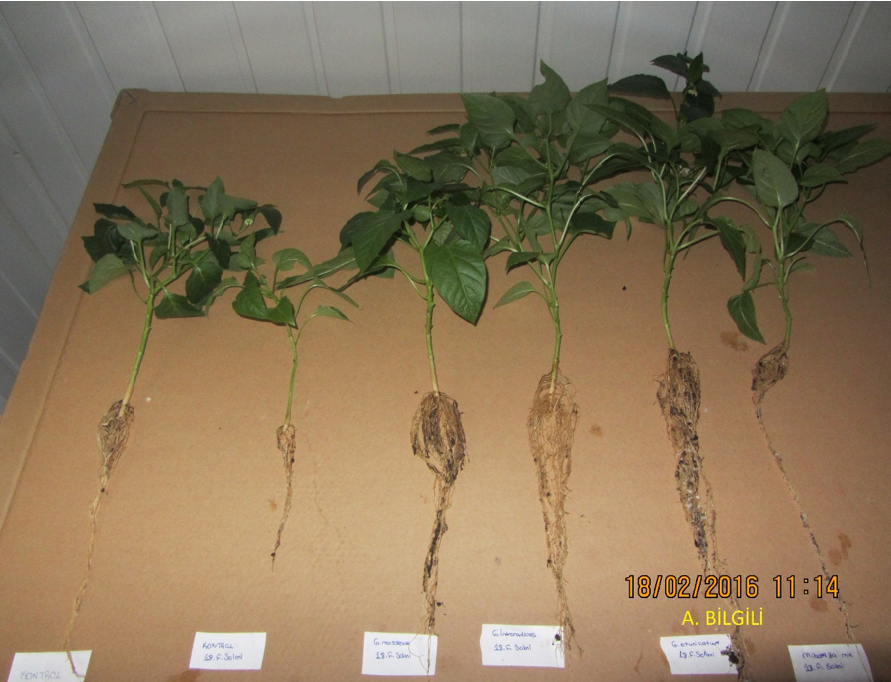

Supplement: Supplemental Information 21 [file peerj-13-18438-s021.png]

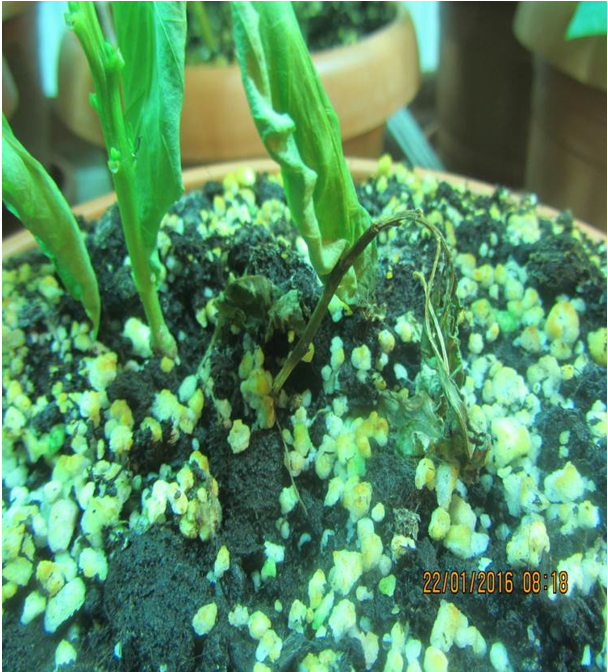

Supplement: Supplemental Information 22 [file peerj-13-18438-s022.png]

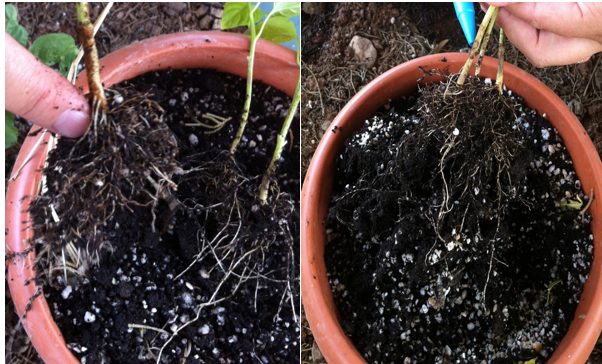

Supplement: Supplemental Information 23 [file peerj-13-18438-s023.png]

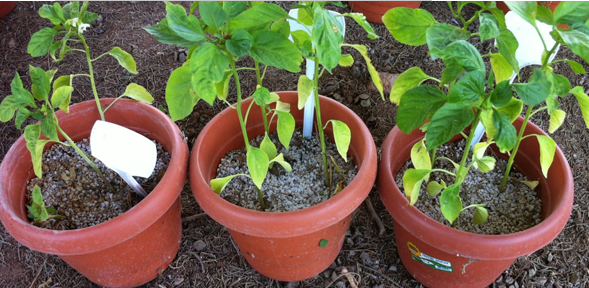

Supplement: Supplemental Information 24 [file peerj-13-18438-s024.png]
